# Regulation of functional *KCNQ1OT1* lncRNA by β-catenin

**DOI:** 10.1038/srep20690

**Published:** 2016-02-12

**Authors:** Naohiro Sunamura, Takahito Ohira, Miki Kataoka, Daigo Inaoka, Hideyuki Tanabe, Yuji Nakayama, Mitsuo Oshimura, Hiroyuki Kugoh

**Affiliations:** 1 Department of Biomedical Science, Institute of Regenerative Medicine and Biofunction, Graduate School of Medical Science, Tottori University, 86 Nishi-Cho, Yonago, Tottori 683-8503, Japan; 2 Department of Evolutionary Studies of Biosystems Science, School of Advanced Sciences, SOKENDAI (The Graduate University for Advanced Studies), Shonan Village, Hayama, Kanagawa 240-0193, Japan; 3 Division of Functional Genomics, Research Center for Bioscience and Technology, Tottori University, 86 Nishi-Cho, Yonago, Tottori 683-8503, Japan; 4 Chromosome Engineering Research Center, Tottori University, 86 Nishi-Cho, Yonago, Tottori 683-8503, Japan

## Abstract

Long noncoding RNAs (lncRNAs) have been implicated in many biological processes through epigenetic mechanisms. We previously reported that *KCNQ1OT1*, an imprinted antisense lncRNA in the human *KCNQ1* locus on chromosome 11p15.5, is involved in *cis*-limited silencing within an imprinted *KCNQ1* cluster. Furthermore, aberration of *KCNQ1OT1* transcription was observed with a high frequency in colorectal cancers. However, the molecular mechanism of the transcriptional regulation and the functional role of *KCNQ1OT1* in colorectal cancer remain unclear. Here, we show that the *KCNQ1OT1* transcriptional level was significantly increased in human colorectal cancer cells in which β-catenin was excessively accumulated in the nucleus. Additionally, overexpression of β-catenin resulted in an increase in *KCNQ1OT1* lncRNA-coated territory. On the other hand, knockdown of β-catenin resulted in significant decrease of *KCNQ1OT1* lncRNA-coated territory and an increase in the mRNA expression of the *SLC22A18* and *PHLDA2* genes that are regulated by *KCNQ1OT1*. We showed that β-catenin can promote *KCNQ1OT1* transcription through direct binding to the *KCNQ1OT1* promoter. Our evidence indicates that β-catenin signaling may contribute to development of colorectal cancer by functioning as a novel lncRNA regulatory factor via direct targeting of *KCNQ1OT1*.

A number of long non-coding RNAs (lncRNAs) have recently been identified through rapid advances in high-throughput analyses of transcriptomes^1^,^2^. Several of these lncRNAs are antisense lncRNAs, which are transcribed from the antisense strand of transcriptional units^3^. Additionally, evidence has been provided that antisense lncRNAs, such as the antisense noncoding RNA in the *INK4A* locus (*ANRIL*), the HOX transcript antisense RNA (*HOTAIR*) and the *KCNQ1* opposite strand/antisense transcript 1 (*KCNQ1OT1*, also known as *LIT1*) can epigenetically regulate the expression of neighboring genes in *cis* or of distant genes in *trans*^4^,^5^,^6^. In contrast, alteration of those lncRNAs directly leads to mis-regulation of the lncRNA target genes, which ultimately resulting in the contraction of various diseases including cancers^4^,^5^,^7^. These findings suggest that epigenetic regulation of gene expression by antisense lncRNA is closely associated with and contributes to many cellular functions. However, the molecular mechanism by which transcriptional regulatory factors control antisense lncRNA remains unclear.

We previously identified the antisense lncRNA *KCNQ1OT1* as an imprinted gene at the *KCNQ1* cluster on human chromosome 11p15.5 by using a novel *in vitro* system that was developed for the screening of imprinted genes using human monochromosomal hybrids^6^. *KCNQ1OT1* is stably accumulated at its own gene region and that some imprinted genes on the *KCNQ1* cluster, which lie outside of the *KCNQ1OT1* lncRNA transcriptional domain throughout the cell cycle, suggesting that *KCNQ1OT1* may play a significant role as a regulatory factor at a specific domain such as at an imprinted cluster^6^,^8^,^9^. Thus, these previous studies indicated that *KCNQ1OT1* lncRNA may control gene expression by accumulation at a sub-chromosomal region in a way that resembles X chromosome inactivation (XCI) by *XIST* RNA^10^. In contrast, it was previously reported that colorectal tissues and cancer cell lines harbor aberrations of *KCNQ1OT1* transcription and epigenetic statuses including histone modifications and DNA methylation at the *KCNQ1* cluster^9^. These evidences suggested that *KCNQ1OT1* transcription may be closely related to initiation and/or progression of colorectal cancer. However, as yet little is known regarding the mechanism by which *KCNQ1OT1* lncRNA is regulated and its functional role in cancer development.

The majority of colorectal cancers are driven by aberration of the Wnt/β-catenin signaling pathway^11^. In the absence of Wnt signals, β-catenin that is located in the cytoplasm is degraded by a protein complex consisting of adenomatous polyposis coil (*APC*), axis inhibitor (*AXIN*), casein kinase 1α (*CK1α*) and glycogen synthase kinase 3β (*GSK3-β*)^12^. The β-catenin level in the cytoplasm was shown to be increased in colorectal cancers that have an active Wnt signaling pathway, and this β-catenin eventually translocates to the nucleus, leading to the transcription of target genes such as cell proliferation-associated genes^12^,^13^,^14^. Furthermore, a lncRNA *E2F4* transcription was directly activated by the β-catenin that accumulated in the nucleus in colorectal cancers, resulting in cancer progression^15^. This finding suggested that aberrations in *KCNQ1OT1* lncRNA in colorectal cancers could be caused by an effect of β-catenin activity.

In the present study, we showed that *KCNQ1OT1* transcription in colorectal cancer cell lines is driven by direct binding of β-catenin to its promoter region. Moreover, both *KCNQ1OT1* transcription and the extent of *KCNQ1OT1* lncRNA-coated territory changed remarkably depending on β-catenin activity. These data provided additional novel evidences that the regulation of *KCNQ1OT1* lncRNA by β-catenin signaling may be involved in the multiple processes of colorectal cancer development.

## Results

### The *KCNQ1OT1* transcription level is associated with the amount of nuclear β-catenin in colorectal cancer cell lines

**Figure 1:**
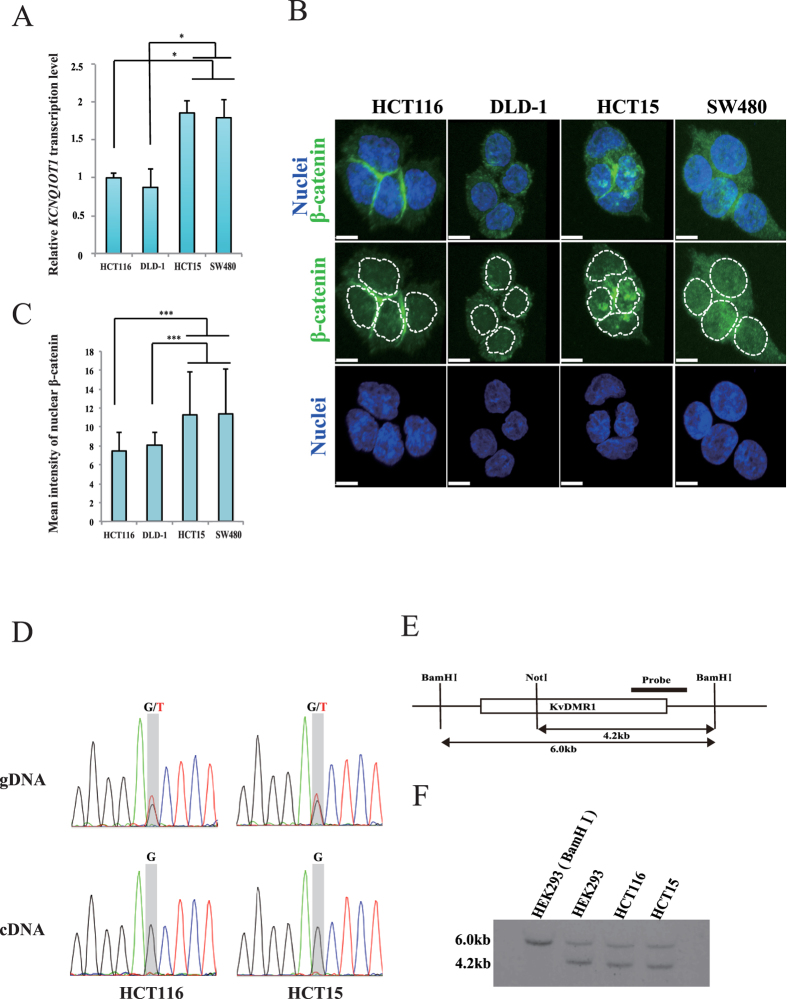
Comparison of the levels of *KCNQ1OT1* transcription and β-catenin protein in colorectal cancer cell lines. The colorectal cancer cell lines HCT116, DLD-1, HCT15 and SW480 were analyzed as follows. (**A**) Relative *KCNQ1OT1* transcription levels were determined using qRT-PCR analysis. Transcription levels were normalized to *GAPDH* mRNA control in the same cell line. The transcription level in HCT116 cells was arbitrarily assigned as 1. Error bars represent means ± S.D. of three independent experiments (*p < 0.05). (**B**) Representative immunofluorescence staining of the expression of β-catenin (green). Nuclei (blue) were stained with DAPI. The dotted lines show individual nuclei. Scale bars represent 10 μm. (**C**) Quantification of nuclear β-catenin. The relative mean intensity of nuclear β-catenin staining of the colorectal cancer cell lines is shown. The graphs represent the average mean intensity of a single nucleus in each cell line (HCT116, n = 51; DLD-1, n = 56; HCT15, n = 53; SW480, n = 59; ***p < 0.001). Error bars represent means ± S.D. (**D**) Sanger sequencing chromatograms of *KCNQ1OT1* genome DNA (gDNA; top) and cDNA (bottom), respectively. (**E**) Schematic representation of the KvDMR1 region is indicated. Black bar shows probe using methylation-sensitive southern hybridization. BamH I and Not I site show digestion site for using methylation-sensitive southern hybridization. (**F**) The 6.0 kb BamHI-digested fragment encompassing the KvDMR1 was digested with Not I, resulting in a 4.2 kb fragment. HEK293 cells were digested with BamH I and observed 6.0 kb digested fragment. The methylation-sensitive southern hybridization indicates statuses of methylation (6.0 kb) and unmethylation (4.2 kb) at the KvDMR1 in HEK293, HCT116 and HCT15 cells. Cropped blot images were used in this figure. Full-length blots and full-length gel image are presented in Supplementary Fig. S2.

We have previously reported that aberration of *KCNQ1OT1* transcription is frequently observed in colorectal cancer tissues and cell lines^9^. Here, we first investigated *KCNQ1OT1* expression status in four colorectal cancer cell lines using quantitative reverse transcription PCR (qRT-PCR). The *KCNQ1OT1* transcription level was increased 1.8-fold in HCT15 and SW480 cells compared to that in HCT116 and DLD-1 cells (Fig. 1A, *p < 0.05).

An aberrant Wnt signaling pathway is implicated in the multistep processes for colorectal cancer development. In particular, nuclear accumulation of the key oncogenic factor β-catenin indicates activation of its target genes, which facilitate cancer promoting functions such as cell proliferation^11^,^12^,^15^. To determine if the *KCNQ1OT1* lncRNA transcription status depends on the amount of β-catenin protein in the nucleus, we performed immunofluorescence staining analysis using a β-catenin antibody and measured the relative intensities of the fluorescence signals of β-catenin in the nuclei of HCT116, DLD-1, HCT15 and SW480 cells (Fig. 1B,C and Supplementary Fig. S1). As shown in Fig. 1B,C, the fluorescence intensity of nuclear β-catenin in HCT15 and SW480 cells was higher than that in HCT116 and DLD-1 cells (***p < 0.001). These results indicated that *KCNQ1OT1* lncRNA transcription levels could be increased by nuclear accumulation of β-catenin in colorectal cancer, suggesting that *KCNQ1OT1* transcription could be regulated by β-catenin activity. Next, we performed single polymorphism nucleotide (SNP) analysis of *KCNQ1OT1* transcript (bdSNP: rs231359) in order to validate the status of the allelic expression of *KCNQ1OT1* in HCT116 and HCT15 cells which exhibit differential distribution of β-catenin to cytoplasm and nuclear compartments of cells. Both the G and T alleles were detected in the genomic DNA. In contrast, only G allele was detected in cDNA samples (Fig. 1D). Furthermore, methylation-sensitive southern hybridization analysis of those cells revealed that the methylated (6.0 kb) and unmethylated (4.2kb) alleles were detected at differentially methylated regions (KvDMR) which play a crucial role in maintenance of the parent-of-origin-specific gene expression pattern^6^ (Fig. 1E,F). In addition, two copies of *KCNQ1OT1* locus are frequently observed in HCT116 and HCT15 cells by DNA fluorescence *in situ* hybridization (DNA-FISH) analysis using a *KCNQ1OT1* specific DNA probe (Supplementary Table S1 online). Thus, these results indicate that *KCNQ1OT1* is monoallelically transcribed in HCT116 and HCT15 cells, suggesting that increase expression levels of *KCNQ1OT1* lncRNA were attributed to an excess of β-catenin into nuclear.

### Overexpression of β-catenin increases *KCNQ1OT1* transcription and expands lncRNA-coated territory in colorectal cancer

**Figure 2:**
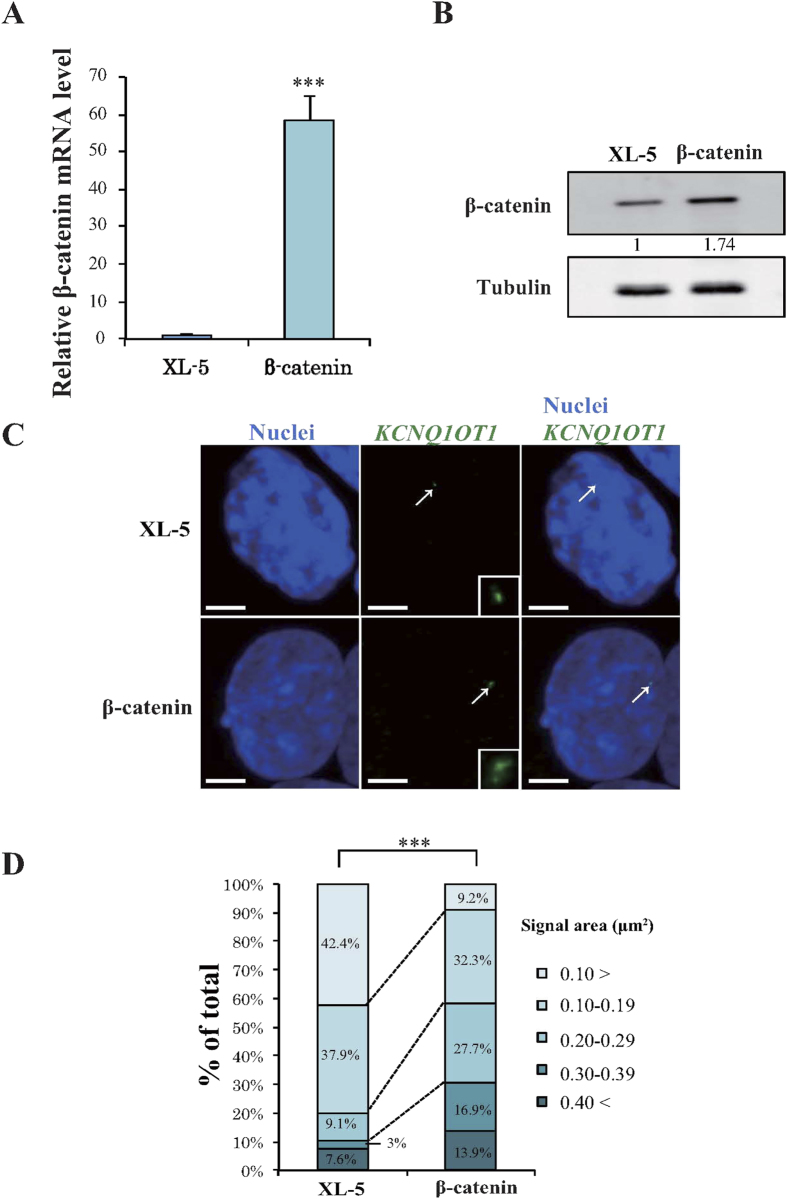
Overexpression of β-catenin enhances transcription and lncRNA-coated territory of *KCNQ1OT1* in HCT116 cells. HCT116 cells were transfected with a β-catenin expression vector or with the control XL-5 vector, and the cells were analyzed 24 h after transfection as follows. (**A**) qRT-PCR analysis of relative β-catenin mRNA expression levels. Expression levels were normalized to *GAPDH* mRNA control. The expression level in HCT116 cells transfected with the XL-5 vector was arbitrarily assigned as 1. Error bars represent means ± S.D. of three independent experiments (***p < 0.001). (**B**) Western blotting analysis of β-catenin protein levels. Band intensities were densitometrically assayed and analyzed using FUJIFILM Multi Gauge software. The protein levels of β-catenin were normalized to the levels of tubulin. The protein level in HCT116 cells transfected with the XL-5 vector was arbitrarily assigned as 1. Cropped blot images were used in this figure. Full-length blots are presented in Supplementary Fig. S3. (**C**) Representative RNA-FISH analysis of *KCNQ1OT1* lncRNA-coated territory (green; arrows) and the insets show enlarged images of *KCNQ1OT1* lncRNA. Nuclei (blue) were stained with DAPI. Scale bars represent 3 μm. (**D**) Quantification of *KCNQ1OT1* lncRNA-coated territory in RNA-FISH analysis. The 100% stacked bar charts indicate the percentage of nuclei displaying the indicated signal area of *KCNQ1OT1* lncRNA-coated territory (β-catenin, n = 65; XL-5, n = 66, ***p < 0.001)

*KCNQ1OT1* lncRNA has been reported to accumulate on its own gene and on targeted regulatory genes. Moreover, the genes on which it accumulates change depending on cell-type and/or developmental stage^8^,^16^,^17^. To further explore the effect of β-catenin on *KCNQ1OT1* transcription, we generated HCT116 cells that transiently overexpressed β-catenin or the control vector (XL-5). Analysis by qRT-PCR and western blotting indicated that the transcription and protein level of β-catenin, respectively, was markedly increased in the β-catenin overexpressing clone compared with that in the control clone (Fig. 2A,B, ***p < 0.001). To investigate whether the *KCNQ1OT1* lncRNA signal in the nucleus, i.e., lncRNA-coated territory, was increased by β-catenin activity, we performed RNA-fluorescence *in situ* hybridization (RNA-FISH). We used a specific probe to detect *KCNQ1OT1* lncRNA, in β-catenin overexpressing HCT116 cells and measured the area of the lncRNA-coated territory. *KCNQ1OT1* lncRNA-coated territory expanded in β-catenin overexpressing HCT116 cells compared with that in the cells transfected with control vector (Fig. 2C). Figure 2D summarizes the score of *KCNQ1OT1* lncRNA-coated territory area in the β-catenin overexpressing and control cells. Compared to the control cells, the clone overexpressing β-catenin displayed a 3.0- and 5.6-fold increase in *KCNQ1OT1* lncRNA-coated territory with a signal area of 0.20–0.29 and 0.30–0.39 μm^2^ respectively, and a 4.6-fold decrease in territory with a signal area of <0.10 μm^2^ (Fig. 2D). To investigate whether endogenous *KCNQ1OT1* transcription was also increased in β-catenin overexpressing HCT116 cells, we performed qRT-PCR analysis. As observed in Supplementary Fig. S3, overexpressing clones of β-catenin resulted in a 1.3-fold increase in *KCNQ1OT1* transcription (*p < 0.05). These results suggested that an increase in *KCNQ1OT1* transcription through β-catenin activity eventually leads to an increase in its lncRNA-coated territory.

### Downregulation of β-catenin results in contraction of *KCNQ1OT1* lncRNA-coated territory and dysregulation of *KCNQ1OT1*-regulated genes in colorectal cancer

**Figure 3:**
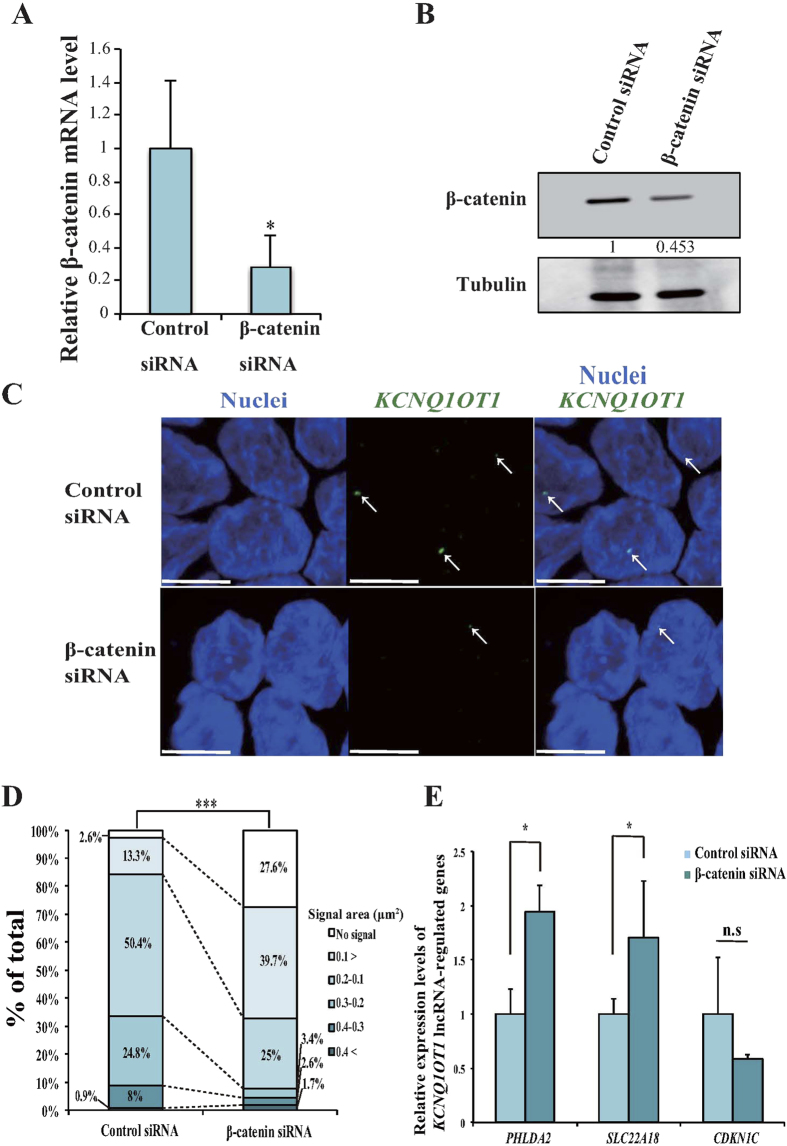
Downregulation of β-catenin reduced transcription, contracted lncRNA-coated territory of *KCNQ1OT1,* and increased in *PHLDA2* and *SLC22A18* expression in HCT15 cells. HCT15 cells were transfected with a β-catenin siRNA or a control siRNA, and the cells were analyzed 72 h after transfection as follows. (**A**) qRT-PCR analysis of relative β-catenin mRNA expression levels. Expression levels were normalized to *GAPDH* mRNA control in each transfected cell type. The expression level in HCT15 cells transfected with control siRNA was arbitrarily assigned as 1. Error bars indicate the means ± S.D. of three independent experiments (*p < 0.05). (**B**) Western blotting analysis of the β-catenin protein level. Band intensities were densitometrically assayed and analyzed using FUJIFILM Multi Gauge software. The protein levels of β-catenin were normalized to the levels of tubulin. The protein level in HCT15 cells transfected with control siRNA was arbitrarily assigned as 1. Cropped blot images were used in this figure. Full-length blots are presented in Supplementary Fig. S4. (**C**) Representative RNA-FISH analysis of *KCNQ1OT1* lncRNA-coated territory (green; arrows). Nuclei (blue) were stained with DAPI. Scale bars indicate 10 μm. (**D**) Quantification of *KCNQ1OT1* lncRNA-coated territory in RNA-FISH analysis. The 100% stacked bar charts indicate the percentage of nuclei displaying the indicated signal area of *KCNQ1OT1* lncRNA-coated territory and no signal (β-catenin siRNA, n = 116; control siRNA, n = 113, ***p < 0.001). (E) qRT-PCR analysis of the relative mRNA expression levels of *KCNQ1OT1* lncRNA-regulated genes. Expression levels were normalized to the level of *GAPDH* mRNA in each transfected cell type. The expression level in HCT15 cells transfected with control siRNA was arbitrarily assigned as 1. Error bars indicate the means ± S.D. of three independent experiments (*p < 0.05).

To further explore the association between β-catenin activity and the area of *KCNQ1OT1* lncRNA-coated territory, we performed knockdown of β-catenin transcription using short interfering RNA (siRNA) in the HCT15 cells that showed the highest amount of nuclear β-catenin protein of the colorectal cancer cell lines tested (Fig. 1C). Knockdown of β-catenin in HCT15 cells reduced its mRNA expression to 35% of that of control cells (*p < 0.05; Fig. 3A). Additionally, the protein level of β-catenin was reduced by 50% (Fig. 3B). To evaluate the effect of decreased β-catenin expression on the extent of *KCNQ1OT1* lncRNA-coated territory in HCT15 cells, we analyzed the *KCNQ1OT1* lncRNA signal pattern with single-cell resolution using RNA-FISH. *KCNQ1OT1* lncRNA-coated territory was contracted by downregulation of β-catenin (Fig. 3C). As summarized in Fig. 3D, compared to control cells, β-catenin siRNA transfected-cells showed a 10.6-fold increase nuclei without *KCNQ1OT1* lncRNA signals and a 2.9-fold increase in *KCNQ1OT1* lncRNA-coated territory area with a signal area of <0.10 μm^2^. Moreover, a 2.0- and 7.2-fold decrease in territory with a signal area of 0.10–0.19 and 0.2–0.29 μm^2^, respectively. These results suggested that a decrease in *KCNQ1OT1* transcription by downregulation of β-catenin caused contraction of the *KCNQ1OT1* lncRNA-coated territory in HCT15 cells.

We previously reported that *KCNQ1OT1* lncRNA accumulates on its own gene region and that the solute carrier family 22 member 18 gene, *SLC22A18*, pleckstrin homology-like domain, family A member 2 gene, *PHLDA2* and cyclin-dependent kinase inhibitor 1C gene, *CDKN1C*, which lie outside of the *KCNQ1OT1* lncRNA transcriptional domain, is regulated by *KCNQ1OT1* lncRNA spreading to other gene regions^6^,^8^. To examine whether reduction in β-catenin activity in the nucleus induces dysregulation of the *SLC22A18, PHLDA2* and *CDKN1C* genes through contraction of *KCNQ1OT1* lncRNA-coated territory, we analyzed the expression level of *SLC22A18, PHLDA2* and *CDKN1C* mRNA in β-catenin siRNA-transfected and control siRNA-transfected HCT15 cells using qRT-PCR. The β-catenin siRNA-transfected HCT15 cells displayed a 1.9- and 1.7-fold increase in *PHLDA2* and *SLC22A18* mRNA levels compared with the control cells, respectively (Fig. 3E, *p < 0.05). In contrast, no remarkable change in the *CDKN1C* mRNA level was observed (Fig. 3E), indicating that the remaining *KCNQ1OT1* lncRNA-coated territory may function on *CDKN1C* adjacent to the *KCNQ1OT1* transcription site. Thus, these results indicated that a decrease in β-catenin activity can cause at least dysregulation of the expression of *KCNQ1OT1*-targeted genes through contraction of *KCNQ1OT1* lncRNA-coated territory from *SLC22A18* to *PHLDA2* locus, suggesting that fluctuation in *KCNQ1OT1* lncRNA by the accumulation of nuclear β-catenin may play a role as an important step in colorectal cancer development.

### *KCNQ1OT1* transcription is directly regulated by β-catenin in colorectal cancer

**Figure 4:**
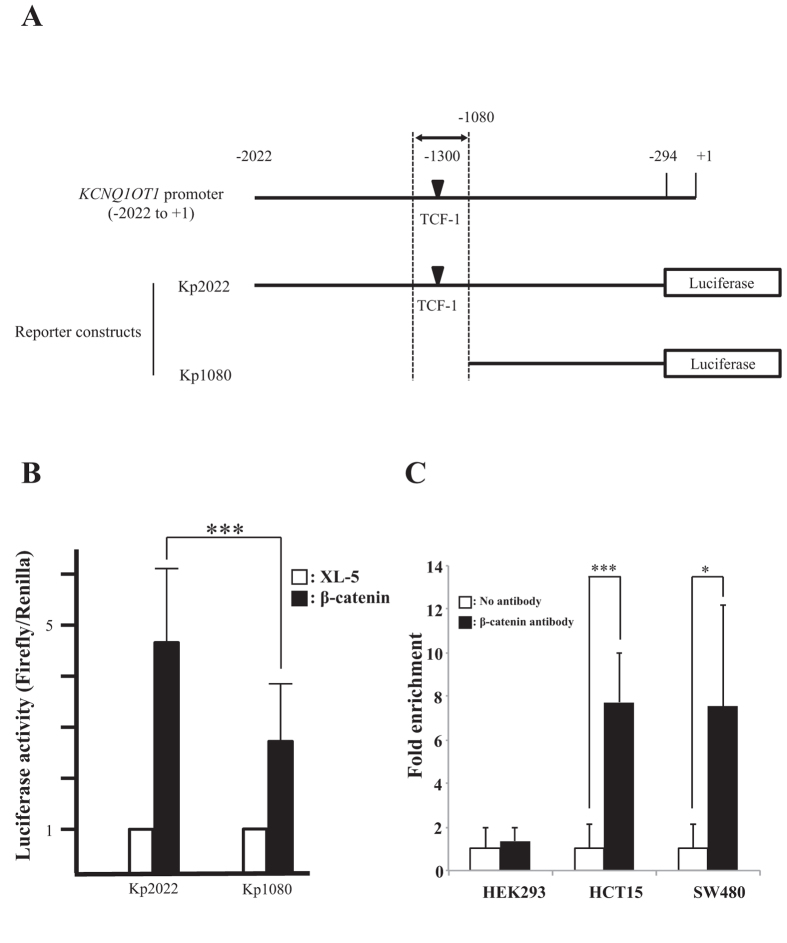
β-catenin directly regulated *KCNQ1OT1* promoter activity *in vivo* in colorectal cancer cells via binding to a TCF-1 site in the *KCNQ1OT1* promoter. (**A**) Schematic representation of the *KCNQ1OT1* promoter region and the reporter constructs used for the luciferase assay. The region of the *KCNQ1OT1* promoter shown is from the transcription start site (TSS, + 1) to −2022. The predicted β-catenin binding site (TCF-1, black inverted triangle) is located −1300 up-stream from the TSS. Arrows indicate the PCR amplification region −1211 to −1351 for the ChIP assay. The two luciferase reporter vectors used in the luciferase assay are indicated. One reporter contains the *KCNQ1OT1* promoter region from −294 to −2022 (Kp2022). The other reporter contains a truncated promoter in which the TCF-1 region was deleted (Kp1080). (**B**) These reporter or control vectors were co-transfected with into HCT116 cells. 24 h later, luciferase activity was measured. Renilla luciferase values were normalized to total protein concentration. Luciferase activity in HCT116 cells transfected with Kp2022 and the XL-5 vector or Kp1080 and the XL-5 were arbitrarily set at 1, respectively. Data are presented as the means ± S.D. of six independent experiments (***p < 0.001). (**C**) The ChIP assay of β-catenin binding to the TCF site in the *KCNQ1OT1* promoter in the human embryonic kidney (HEK293) cell line and the colorectal cancer cell lines HCT15 and SW480. The assay was performed with or without β-catenin antibody. DNA was recovered from immunoprecipitated and nonimmunoprecipitated (input) chromatin and analyzed by qRT-PCR. The fold enrichment of target sequence in precipitated DNA compared with input DNA was calculated by comparison of the threshold cycle value of the sample of precipitated DNA with the standard curve generated from those of input DNA. Data are presented as the means ± S.D. of three independent experiments (***p < 0.001 and *p < 0.05).

To investigate whether β-catenin regulates *KCNQ1OT1* lncRNA transcription through association with the *KCNQ1OT1* promoter and regulation of promoter activity, we investigated the presence of TCF consensus binding sites, which are known to be important for β-catenin regulation of other promoters, on the promoter region of *KCNQ1OT1*. We first selected a TCF binding site that is predicted to be located closest to the transcription start site on *KCNQ1OT1,* based on the search software (MatInspector) for transcription factor binding sites that supports Genomatix software. Second, we constructed human *KCNQ1OT1* promoter-luciferase reporter plasmids; plasmid Kp2022 contained a 2020-bp fragment from within the *KCNQ1OT1* promoter region that included this TCF binding site, and plasmid Kp1080 contained a truncated fragment of the 5′ region of the *KCNQ1OT1* promoter that did not include this TCF binding site (Fig. 4A). We then examined the effect of co-transfection of a β-catenin expressing vector or the control XL-5 vector on the transcriptional activity of these *KCNQ1OT1* promoters by measurement of luciferase activity in HCT116 cells. *KCNQ1OT1* promoter activity in the β-catenin/Kp1080 transfected cells, whose reporter lacked the selected TCF binding site, was only 40.6% that of the β-catenin/ Kp2022 transfected cells, whose reporter included the TCF binding site (Fig. 4B, ***p < 0.001). This result suggested that β-catenin regulated *KCNQ1OT1* lncRNA transcription through an effect on the *KCNQ1OT1* promoter.

To determine whether β-catenin directly binds to a TCF site in the *KCNQ1OT1* promoter, we performed chromatin immunoprecipitation (ChIP) analysis. In this assay, crosslinking of β-catenin to the TCF binding site in the *KCNQ1OT1* promoter in cells of the colorectal cancer cell lines HCT15 and SW480 was assayed (Fig. 4C). The result indicated that β-catenin directly binds to a TCF-1 site on the *KCNQ1OT1* promoter. Thus, *in vivo,* β-catenin can directly regulate *KCNQ1OT1* transcription suggesting that the regulation of *KCNQ1OT1* lncRNA by β-catenin signaling may be involved in the multiple processes of colorectal cancer development.

## Discussion

We reported here that the β-catenin directly regulated *KCNQ1OT1* lncRNA transcription through targeting its promoter region. Moreover, β-catenin affected the extent of *KCNQ1OT1* lncRNA-coated territory in a dose-dependent manner, resulting in at least dysregulation of *KCNQ1OT1*-targeted genes in colorectal cancer.

Differentially methylated regions (DMR) associated with imprinted clusters function as important regions for maintenance of the parent-origin-specific gene expression pattern, which is known as the imprinting control region (ICR). The ICR of the *KCNQ1* cluster harbors the *KCNQ1OT1* promoter region^7^. A previous study reported that the transcription factor NF-Y directly binds to the *Kcnq1* ICR, resulting in loss of *Kcnq1ot1* transcription^18^. NF-Y mediated *Kcnq1ot1* transcription thereby plays a crucial role in regulating the bidirectional silencing of neighboring imprinted genes^18^. Here, we found that β-catenin also binds to the proximal region of the ICR within the *KCNQ1OT1* promoter, and affects the expression of *KCNQ1OT1* and of *KCNQ1OT1*-targeted genes (Figs 2,3 and 4). In addition, an excess of β-catenin protein in cells is a key factor for the development of colorectal cancer. Indeed, the accumulation of β-catenin in the nucleus is frequently observed in colorectal cancer cell lines (Fig. 1)^13^. It is therefore likely that at least two distinct *KCNQ1OT1* regulatory factors exist at the *KCNQ1* ICR.

The lncRNA-mediated gene silencing behavior of *KCNQ1OT1* lncRNA resembles that of *Xist* RNA in terms of chromatin association and *cis*-limited epigenetic silencing^8^. *Xist* RNA is essential for X chromosome inactivation (XCI). *Xist* RNA accumulates and spreads along the X chromosome, which expresses its RNA, and then recruits gene silencing complexes that include histone methyltransferases and the polycomb group proteins *Eed*/*Ezh2*/*Suz12*, which ultimately establishes XCI by induction of heterochromatin formation^10^. Furthermore, we found that genes such as *Jarid1c* or *Utx* that escape from XCI are always located at the periphery or outside of the *Xist* RNA-coated territory in C2C12 normal mouse myoblast cells^19^. In contrast, during the stage of prenatal development, the mouse *Kcnq1ot1* lncRNA-coated territory area in placental derived cells is largely expanded compared to that of embryonic cells^17^,^20^. This expansion is because the *Kcnq1ot1* lncRNA-silencing target locus differs in the placenta and the embryo^17^,^20^. Thus, these evidences suggest that expansion of *cis*-limited lncRNA-coated territory is coordinated with the silencing behavior of its coated-genes. In the present study, we demonstrated that the mRNA expression of *SLC22A18* and *PHLDA2*, which are regulated by *KCNQ1OT1* lncRNA spreading, was increased by the knockdown of β-catenin in colorectal cancer cells (Fig. 3). Therefore, aberration of the extent of the *KCNQ1OT1* lncRNA-coated territory that is caused by an excess of nuclear β-catenin may play a significant role in the process of cancer development. However, We do not rule out the possibility that aberration of *KCNQ1OT1* lncRNA-coated territory may significantly affect genes and microRNAs involved in cancer initiation or progression rather than the expression of specific genes that located on chromosome 11, which would ultimately lead to disruption of the balance of gene expression in a whole cell.

Aberration of lncRNA transcription including that of *lncRNA-p21*, *KCNQ1OT1*, colorectal cancer associated transcript 1 long isoform (*CCAT1-L*), *HOTAIR, E2F4* antisense transcript and metastasis associated lung adenocarcinoma transcript 1 (*MALAT1*) has been observed in colorectal cancer that harbors alteration of Wnt/β-catenin signaling pathways^9^,^15^,^21^,^22^,^23^,^24^. In this respect it is interesting that dysregulation of the *E2F4* antisense transcript and of *MALAT1* transcription, which cause accumulation of β-catenin in the nucleus, strongly contributes to the development of colorectal cancer^15^,^24^. We demonstrated that β-catenin directly activates *KCNQ1OT1* transcription through binding to its promoter region (Figs 2,3 and 4). These combined evidences indicated that aberrations of some lncRNAs are strongly associated with Wnt/β-catenin signaling pathways that contribute to colorectal cancer progression, and which are important during the multistep processes of neoplastic development.

We found that accumulation of nuclear β-catenin induced dysregulation of *KCNQ1OT1* transcription in colorectal cancer cells (Figs 2 and 3). This phenomenon has also been observed in various other cancers including melanoma, ovarian carcinoma and gastric cancer^25^,^26^,^27^. Thus, these findings strongly support the hypothesis that dysregulation of *KCNQ1OT1* transcription by nuclear β-catenin may be involved in the development of various cancers.

In conclusion, our study demonstrated that excessive nuclear β-catenin causes aberration in the extent of *KCNQ1OT1* lncRNA-coated territory, suggesting that a change in its lncRNA-coated territory profile may strongly contribute to the multistep processes that lead to the establishment of malignant colorectal cancer. However, the mechanism by which gene transcription is influenced by aberration of the extent of *KCNQ1OT1* lncRNA-coated territory remains to be clarified. Further studies aimed at identification of *KCNQ1OT1* lncRNA-targeted genes and the factors that maintain and regulate *KCNQ1OT1* lncRNA-coated territory will be necessary in order to clarify the significance of the regulation of the extent of *KCNQ1OT1* lncRNA-coated territory through an oncogenic signaling pathway such as Wnt/β-catenin in cancer development.

## Materials and Methods

### Cell lines and Cell culture

HCT116, DLD-1, HCT15, SW480 and HEK293 cells were purchased from the ATCC (#CCL-247, #CCL-221, #CCL-225, #CCL-228, #CRL-1573, respectively). HCT116, DLD-1, HCT15 and HEK293 cells were cultured in Dulbecco’s modified Eagle’s medium (DMEM; Sigma-Aldrich, St. Louis, MO, USA) supplemented with 10% fetal bovine serum (FBS; HyClone, Logan, UT, USA). SW480 cells were cultured in Leibovitz’s L-15 medium (Thermo Fisher Scientific, Gibco Cell Culture, Rockford, IL, USA) supplemented with 10% FBS.

### Plasmid transfection and siRNA-mediated knockdown

Cells were transfected with plasmid or siRNA using Lipofectamine 2000 (Thermo Fisher Scientific, Invitrogen). For overexpression of β-catenin, 1 × 10^6^ HCT116 cells were seeded in each well of six wells plates and were transfected 24 h after seeding with 4 μg of β-catenin expression vector or XL-5 vector. Both the β-catenin expression vector and XL-5 were purchased from OriGene (#SC107921, OriGene, Rockville, MD, USA). For knockdown of β-catenin, 5 × 10^5^ HCT15 cells were seeded in each well of 6 plates and were transfected 72 h after seeding with 100 pmol of β-catenin siRNA (siRNA ID 42816, Thermo Fisher Scientific, Ambion) or control (#SN-1003, Bioneer, Seoul, Korea).

### Luciferase assay

The Luciferase assay was performed as described previously^28^. In brief, a segment of the human *KCNQ1OT1* promoter region containing the predicted binding site for TCF-1 based on the Transcription Element Search System (http://www.cbil.upenn.edu/tess), Genomatix GEMS Lancher (http://www.genomatix.de) and Genetyx Ver.10 software (Genetyx, Tokyo, Japan) was PCR-amplified from cDNA and inserted into a BglII/Acc65I-digested luciferase reporter vector pGL3-basic (Promega, Madison, WI, USA). The PCR primer sets were designed to amplify the upstream *KCNQ1OT1* promoter region from -2022 to -294. The PCR primer sets used were: forward primer: 5′-GGGGTACCCCAGGTGACAAGGTGCAGGCGC and reverse primer: 5′-ACAGAGTTCCTCGTTGGGAGCTTGAAGATCTTC. For the truncated luciferase reporter construct without the TCF-1 site (Kp1080), deletion of the upstream *KCNQ1OT1* promoter region −2022 from −1080 was performed using PCR-based site-directed mutagenesis (Toyobo, Osaka, Japan). Kp1080 was PCR-amplified from the Kp2022 luciferase reporter construct according to the manufacturer’s protocol. The PCR primer sets used were: forward primer: 5′-CGGAGGTGGGAATCCCCGTTG, and reverse primer: 5′-GGGGTACCTATCGATAGAGAAATG.

### Methylation-sensitive southern hybridization

Methylation-sensitive southern hybridization was performed using DIG High Prime DNA Labeling and Detection Kit (Roche Applied Science, Penzberg, Germany) according to the manufacturer’s instructions. In brief, 20 μg of genome DNA from normal and colorectal cancer cell lines were digested with BamHI and NotI overnight, resolved by gel electrophoresis, and transferred to High bond N + (GE Healthcare, Piscataway, NJ, USA). The PCR products were labeled by Digoxigenin-11-dUTP and used as probe. The PCR primer sets used were: forward primer: 5′- TCTCTCTGGGAGGGTTTGAA and reverse primer: 5′- TTACTTCGCCCCCTAATTCCT. Immunoreactive bands were analyzed using a Luminescent Image Analyzer LAS-4000 (Fujifilm, Tokyo, Japan).

### Western blotting

Western blotting was performed as described previously^28^. The membranes were blotted with a rabbit monoclonal antibody against the human β-catenin antigen (1:2000, #8480, Cell Signaling Technology, Tokyo, Japan), or with a rabbit polyclonal antibody against the human α/β-tubulin antigen (1:2000, #2148, Cell Signaling Technology) and the appropriate standard peroxidase-labeled anti-rabbit IgG secondary antibody was used according to the manufacturer’s instructions (GE Healthcare). Immunoreactive bands were analyzed using a Luminescent Image Analyzer LAS-4000 (Fujifilm) and β-catenin protein levels were quantified using Multi Gauge V3.0 software (Fujifilm).

### SNP analysis

Search of allelic specific SNP on *KCNQ1OT1* locus was used dbSNP website (http://www.ncbi.nlm.nih.gov/SNP/) and PCR products spanning SNP from colorectal cancer cell lines were sequenced (Eurofins Genomics, Tokyo, Japan). The PCR primer sets were: forward primer: 5′-GGGTAGGCTGGTCACGTTTA and reverse primer: 5′-AGTCCCCTGTAGATTCTGGG. The sequence data was analyzed using Finch TV (http://www.geospiza.com).

### ChIP assay

ChIP assay was performed as described previously^9^. A mouse monoclonal antibody against human β-catenin (#610153, BD Japan, Tokyo, Japan) was used for the ChIP assay. Precipitated DNA was amplified using an Applied Biosystems StepOne thermal cycler system and a SYBR green PCR kit (Thermo Fisher Scientific, Applied Biosystems) and the following TCF-1 site-specific detection primers: forward; 5′- GGTTCTGAGTCCGCGCTATT, and reverse; 5′- GGATTCCCACCTCCGATCCT.

### Immunofluorescence staining

Cells (2.4 × 10^4^) were seeded on 24 × 60 mm micro cover glass and were incubated at 37 °C for 24 h. The cells were then fixed with 4% paraformaldehyde at room temperature for 10 min. After two cold PBS washes, the cells were treated 0.5% saponin (Sigma-Aldrich) and 0.5% Triton X-100 (Sigma-Aldrich) at room temperature for 20 min and were then kept in 20% glycerol (Wako, Osaka, Japan) /PBS for 2 h. Subsequently, the cells were passed six times through liquid nitrogen. After two PBS washes, the cells were blocked with 5% bovine serum albumin (Sigma-Aldrich) in PBS (5% BSA/PBS). Rabbit monoclonal antibody against the human β-catenin antigen diluted to 1:500 in 3% BSA/PBS was applied to the cells. The cells were washed twice in PBS with 0.05% Tween-20 (PBST; Sigma-Aldrich), followed by addition of the secondary antibody Alexa Fluor 488 goat-anti-rabbit IgG (Thermo Fisher Scientific, Invitrogen) diluted to 1:600 in 3% BSA/PBS. After a PBST wash, the cells were stained with DAPI (Vector Laboratories, Burlingame, CA). Immunofluorescence staining was observed by using the confocal microscope LSM780 (Carl Zeiss, Oberkochen, Germany). Intensity was measured using the ZEN 2010 software (Carl Zeiss). Note that all Immunofluorescence staining experiments were performed in parallel and, for detection, exposure and interval time was kept the same in several experiments.

### RNA-FISH

RNA-FISH analysis was performed as previously described^29^. Note that micro cover glass seeded cells were not heat-denatured in order to avoid hybridization of probes to genomic DNA. Probes were labeled with DNP-11-dUTP (Perkin Elmer Japan, Kanagawa, Japan) using a Nick translation Mix (Roche Applied Science). Anti-DNP-rabbit IgG (Sigma-Aldrich) and Alexa Fluor 488 goat-anti-rabbit IgG (Thermo Fisher Scientific, Invitrogen) were diluted to 1:500 and used to detect DNP-labeled probes. The U90095 P1-derived artificial chromosome (PAC) genomic probe was used for RNA-FISH. The U90095 PAC was obtained from BACPAC Resource Center (BPRC) at Children’s Hospital Oakland Research Institute (CHORI). Signals of the *KCNQ1OT1* transcript were detected using the confocal microscope LSM780 (Carl Zeiss). Images were minimally enhanced for brightness and contrast to resemble that which was seen by eye through the microscope. In setting the confocal microscope, the objective lens used was the Plan-Apochromat 63x/1.40 Oil DIC M27, and the lasers used were 405 nm (DAPI) and 488 nm (Alexa 488). The image sizes were: x, 512; y, 512; z, 13, and 12-bit. Area was measured using the ZEN 2010 software (Carl Zeiss). Note that all RNA-FISH experiments were performed in parallel and, for detection, exposure and interval time was kept the same in several experiments.

### DNA-FISH

DNA-FISH analysis was performed as previously described^30^. Metaphase images were captured digitally with a cooled CCD camera equipped with an ISIS (Carl Zeiss) and then copy number of *KCNQ1OT1* locus were counted.

### qRT-PCR

RNA isolation and reverse transcriptase (RT)-PCR were performed as described previously^28^. *KCNQ1OT1* transcription, and β-catenin and *SLC22A18* mRNA expression were detected using qRT-PCR. *KCNQ1OT1* transcription, and β-catenin and *SLC22A18* mRNA expression, were analyzed using the following specific primers. *KCNQ1OT1*: forward, 5′-CTTTGCAGCAACCTCCTTGT; reverse, 5′-TGGGGTGAGGGATCTGAA. β-catenin: forward, 5′-TCTGATAAAGGCTACTGTTGGATTGA; reverse, 5′-TCACGCAAAGGTGCATGATT; *SLC22A18:* forward, 5′-CATCTTGCTTACCTACGTGCTG; reverse, 5′-CCCAGTTTCCGAGACAGGTA. *PHLDA2*: forward, 5′- TCCAGCTATGGAAGAAGAAGC

; reverse, 5′- GTGGTGACGATGGTGAAGTACA. *CDKN1C*: forward, 5′- CTCCGCAGCATCCACGAT; reverse, 5′- GGTGCGCACTAGTACTGGGA. cDNA was amplified using an Applied Biosystems StepOne thermal cycler system and a SYBR green PCR kit (Thermo Fisher Scientific, Applied Biosystems). The mRNA level was normalized to human *GAPDH* mRNA (PCR primers: forward, 5′-AGCCACATCGCTCAGACAC; reverse, 5′-GCCCAATACGACCAAATCC).

### Statistical Analysis

Data from more than two separate experiments are presented as means ± S.D. Significance was established at *P*-values less than 0.05 using an unpaired Two-tailed Student’s *t* test. In RNA-FISH analyses, we used the Chi-squared test to determine whether there was any significant difference in the distribution of area ratio between control cells and β-catenin expressing plasmid or siRNA transfected-cells.

## Additional Information

**How to cite this article**: Sunamura, N. *et al.* Regulation of functional *KCNQ1OT1* lncRNA by β-catenin. *Sci. Rep.* **6**, 20690; doi: 10.1038/srep20690 (2016).

## Supplementary Material

Supplementary Information
