# Supplementary material for: Regulation of functional KCNQ1OT1 lncRNA by β-catenin
Source: Sci Rep. 2016 Feb 12;6:20690. doi: 10.1038/srep20690 (PMC4751614; doi:10.1038/srep20690)
Supplement: Supplementary Information [file srep20690-s1.pdf]

Supplementary information

Regulation of functional *KCNQ1OT1* lncRNA by β-catenin

Naohiro Sunamura[1], Takahito Ohira[1], Miki Kataoka[1], Daigo Inaoka[1], Hideyuki Tanabe[2], Yuji Nakayama[3], Mitsuo Oshimura[4] and Hiroyuki Kugoh[1, 4*]

[1]Department of Biomedical Science, Institute of Regenerative Medicine and Biofunction, Graduate School of Medical Science, Tottori University, 86 Nishi-Cho, Yonago, Tottori 683-8503, Japan

[2]Department of Evolutionary Studies of Biosystems Science, School of Advanced Sciences, SOKENDAI (The Graduate University for Advanced Studies), Shonan Village, Hayama, Kanagawa 240-0193, Japan

[3]Division of Functional Genomics, Research Center for Bioscience and Technology, Tottori University, 86 Nishi-Cho, Yonago, Tottori 683-8503, Japan

[4]Chromosome Engineering Research Center, Tottori University, 86 Nishi-Cho, Yonago, Tottori 683-8503, Japan

*Correspondence should be addressed to H.K. (kugoh@med.tottori-u.ac.jp).

Department of Biomedical Science, Institute of Regenerative Medicine and Biofunction, Graduate School of Medical Science, Tottori University, 86 Nishi-Cho, Yonago, Tottori 683-8503, Japan,

Telephone number: +81-859-38-6208. Fax number: +81-859-38-6210.

Supplementary Figure S1

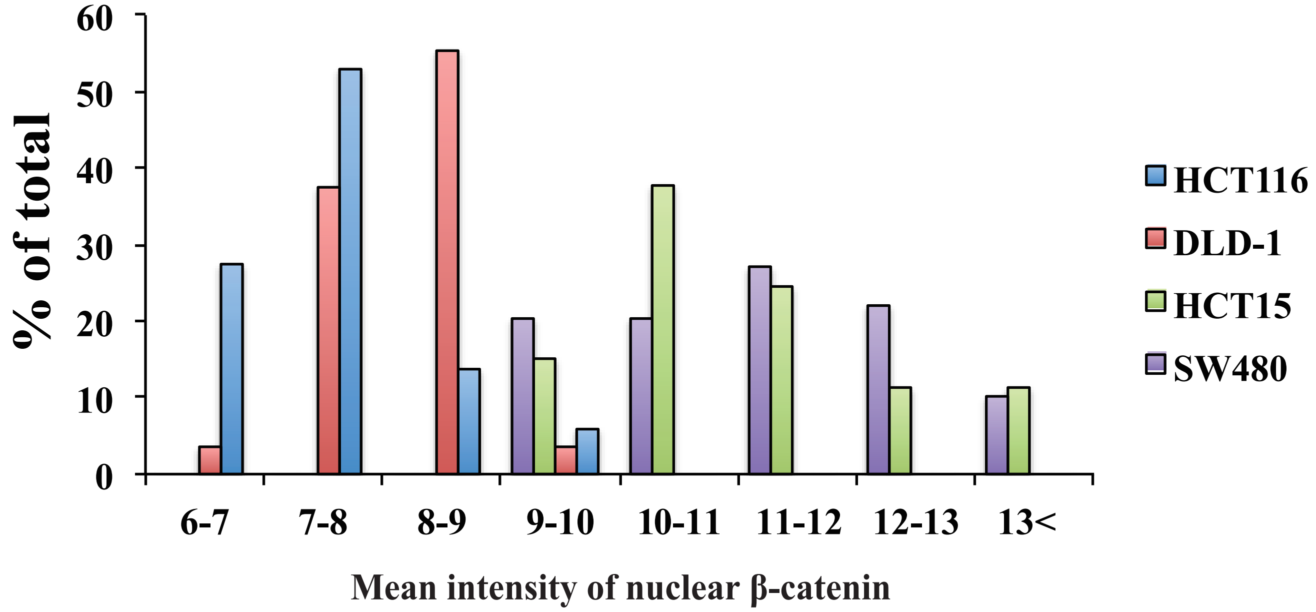

Supplementary Figure S1. Level of nuclear β-catenin protein in colorectal cancer cell lines. Relative mean intensity of nuclear β-catenin protein in colorectal cancer cell lines was evaluated using immunofluorescence staining. The graphs indicate the mean intensity of a single nucleus in each colorectal cell line and nuclei are classified into several groups according to the level of nuclear β-catenin intensity.

Supplementary Figure S2

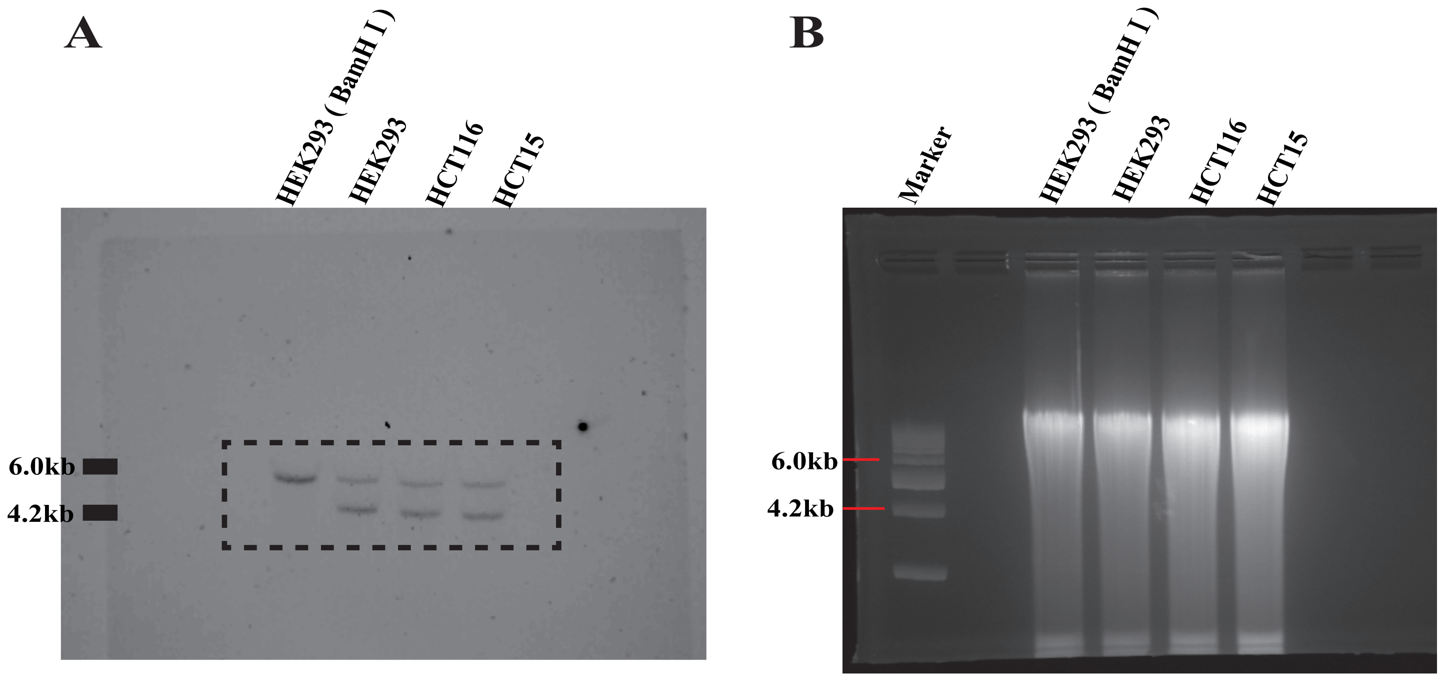

Supplementary Figure S2. Full-length southern blot image (A) and full-gel image (B) shown in Figure 1F. Dotted lines show the cropped areas (see Figure 1F).

Supplementary Table S1

	Copy number of *KCNQ1OT1* (%)				
	1	2	3	4	5
HCT116	14	84	2	0	0
HCT15	0	94	0	6	0

Supplementary Table S1. Copy number of *KCNQ1OT1* locus in HCT116 and HCT15 cells .

Supplementary Figure S3

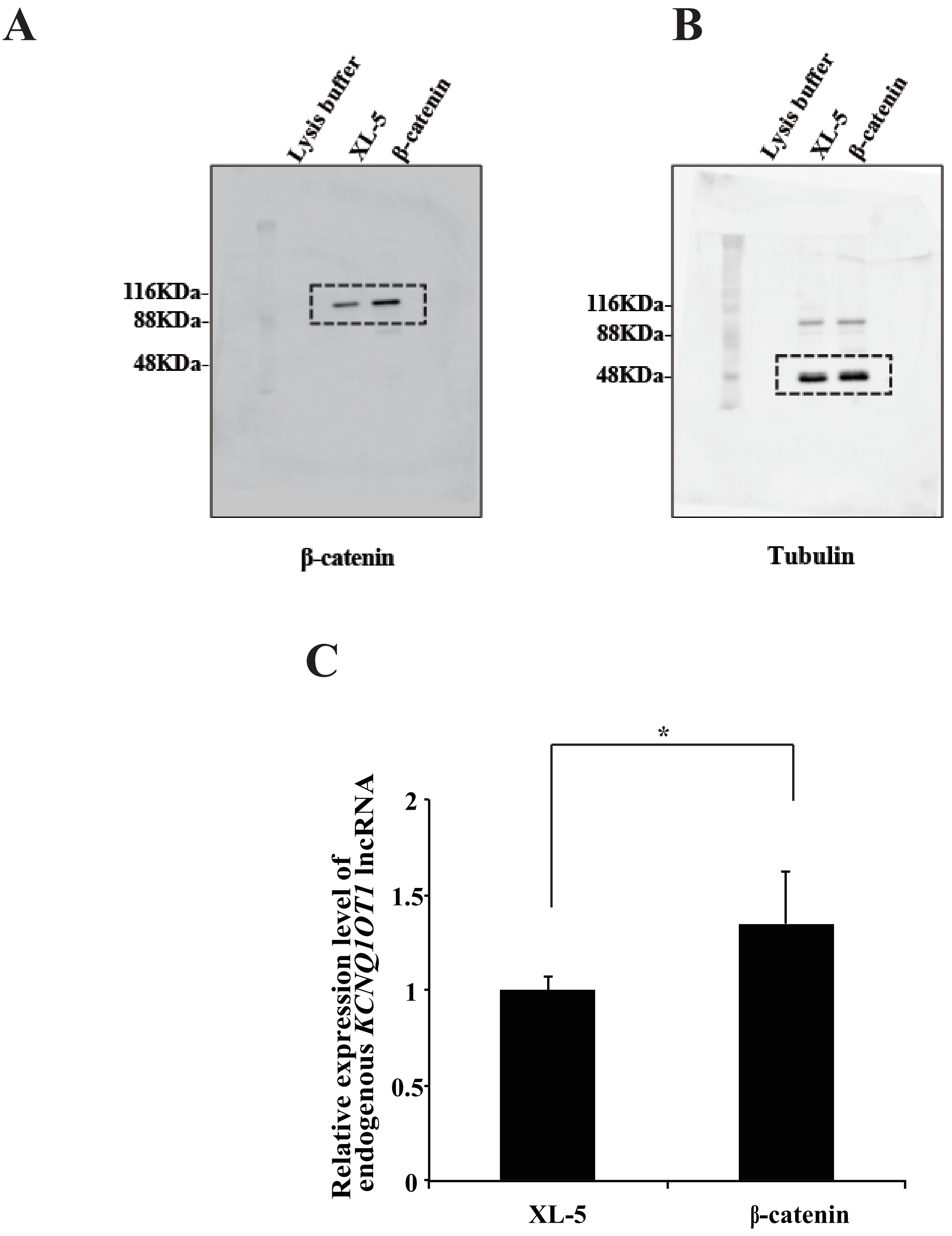

Supplementary Figure S3. Full-length images of the Western blots shown in Figure 2B in the manuscript and qRT-PCR analysis of relative endogenous *KCNQ1OT1* lncRNA transcription under the condition of excess β-catenin.

The full-length Western blot image of (A) the anti-β-catenin antibody blot and (B) the anti-tubulin antibody blot of Figure 2B. Dotted lines show the cropped areas (see Figure 2B). (C) Expression levels were normalized to *GAPDH* mRNA control. The expression level in HCT116 cells transfected with XL-5 vector was arbitrarily assigned as 1. Error bars represent means ± S.D. of four independent experiments (*$p<0.05$).

Supplementary Figure S4

A

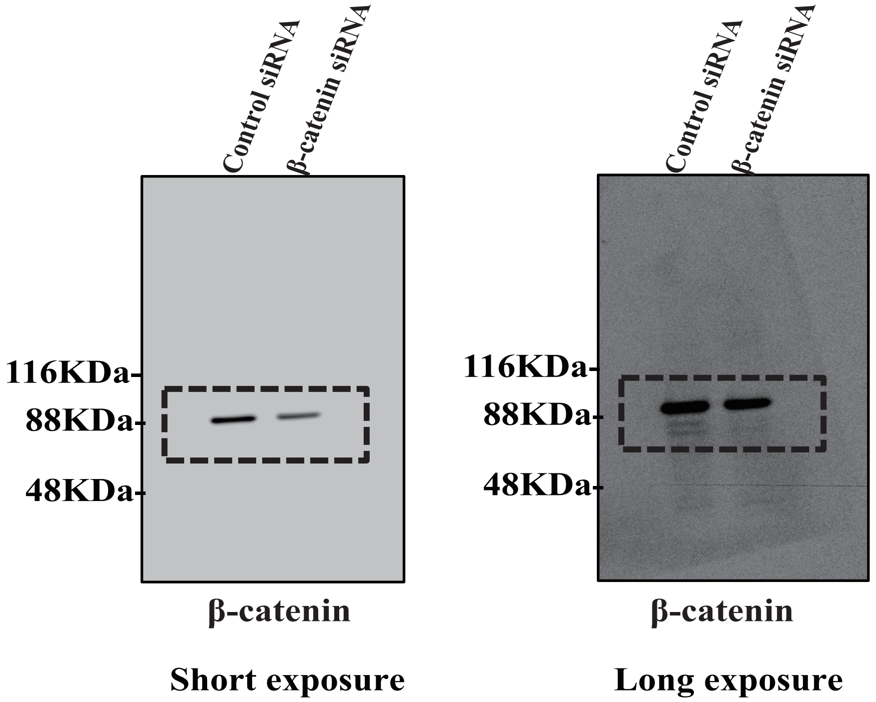

B

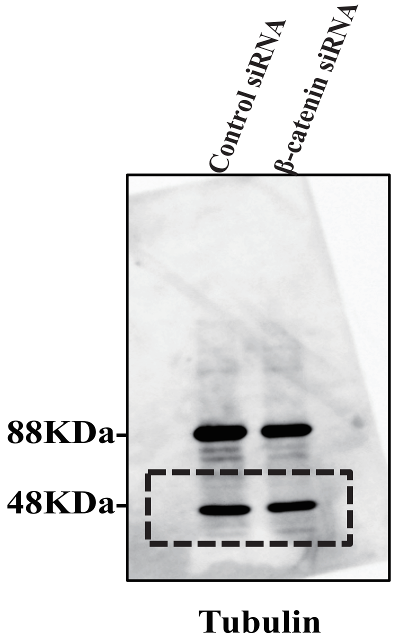

Supplementary Figure S4. Full-length images of the Western blots shown in Figure 3B in the manuscript. The full-length Western blot images of Fig. 3B in the manuscript using (A) an anti-β-catenin antibody and detected with a short (Left) and a long (Right) exposure, and (B) an anti-tubulin antibody. Dotted lines show the cropped areas (see Figure 3B).
